# Effects and safety of virtual reality-based mindfulness in patients with psychosis: a randomized controlled pilot study

**DOI:** 10.1038/s41537-023-00391-8

**Published:** 2023-09-13

**Authors:** Bo Mi Lee, Sung-Wan Kim, Bong Ju Lee, Seung-Hee Won, Yong-han Park, Chae Yeong Kang, Ling Li, Fatima Zahra Rami, Young-Chul Chung

**Affiliations:** 1 Department of Psychiatry, Jeonbuk National University Medical School, Jeonju, 54907 Republic of Korea; 2 Research Institute of Clinical Medicine of Jeonbuk National University-Biomedical Research Institute of Jeonbuk National University Hospital, Jeonju, 54907 Republic of Korea; 3 Department of Psychiatry, Chonnam National University Medical School, Gwangju, 501-746 Republic of Korea; 4 Department of Psychiatry, Inje University Haeundae Paik Hospital, Inje University College of Medicine, Busan, 48108 Republic of Korea; 5 Department of Psychiatry, Kyungpook National University School of Medicine, Daegu, 41944 Republic of Korea; 6 Park’s Psychiatric Clinic, Jeju, Republic of Korea

**Keywords:** Psychosis, Schizophrenia

## Abstract

Virtual reality (VR) technology can be a supporting tool to enhance mindfulness. Recently, many research using VR-based mindfulness (VBM) has been carried out in various psychiatric disorders but not in psychosis. We investigated safety and effects of virtual reality-based mindfulness (VBM) in patients with psychosis as a pilot study. Sixty-four patients were randomly assigned to VBM or to VR control. For VBM, education and meditation videos were provided. For VR control, 3-dimensional natural scenes were shown. Both programs consisted of 8 weekly sessions, each lasting about 30 min. Pre- and post-assessments were performed using the experiences questionnaire (EQ), psychotic symptom rating scales-delusion (PSYRATS-D), PSYRATS-auditory hallucinations (AH), motivation and pleasure scale-self rating (MAP-SR) and etc. The safety questionnaire was also surveyed after 1st and 8th session. Physiological measures such as skin conductance level (SCL), heart rate (HR) and RR interval, were collected during the VR interventions. Limited individuals participated in the safety questionnaire and physiological measures. All the results were presented in mean and standard deviation. We did not observe significant results in group x time interaction and main effects of group and time in the decentering and clinical scales. However, within group comparison showed that patients randomized to VBM showed increased decentering (*p* = 0.029) and decreased amount (*p* = 0.032) and duration of preoccupation (*p* = 0.016) in the PSYRATS-D. For the feelings and motivations about close caring relationships of the MAP-SR, we observed a significant group x time interaction (*p* = 0.027). The frequency of VR sickness was high but its severity was mild. There were significant differences only in HR over time in the VBM group (*p* = 0.01). These results suggest that VBM was not more effective in reducing decentering and psychiatric symptoms than VR control but its adversity was modest.

## Introduction

Virtual reality (VR) involves computer technology that enables the perception of multisensory stimuli within immersive, 3D, complex environments. With VR, patients can practice functioning in familiar settings, which may allow them to develop skills that are more generalizable to real-world situations^1^. As it also provides a realistic and immersive environment tailored to the individual’s needs, patients with mental health problems would feel more engaging and safer trying things in VR. Because of these beneficial features, VR therapy has emerged as a successful solution for a wide range of psychiatric disorders. Studies have shown that VR can be a promising add-on in the treatment, as well as assessment of many psychiatric disorders, such as substance use disorders^2^, specific phobias^3^, posttraumatic stress disorder (PTSD)^4^, stress and anxiety^5–7^ as well as an array of other diagnosis such as Alzheimer’s disease^8^. Although research using this technology in schizophrenia is recent, promising results were shown in the treatment of delusions^9^ and in the evaluation and training of cognitive and social skills^10–13^. All these studies incorporated various components of cognitive-behavioral therapy, exposure therapy or psychoeducation into VR technology.

Mindfulness has been defined as the act of consciously focusing the mind in the present moment without judgment and without attachment to the moment^14^. Mindfulness based interventions (MBIs) have been found effective for the treatment of psychological morbidities and emotional distress in physical and mental illness, including recurrent major depressive disorder, bipolar disorder, chronic pain, stress, anxiety disorders, chronic physical illness, eating disorders, cancer and substance use disorders^15^. The proposed mechanisms of MBI based on the Buddhist psychological model^16^ are acceptance/compassion, attention regulation, ethical practices, nonattachment and nonaversion, and decreased mental proliferation^17^. Historically, clinical literature has warned against the use of meditation with people experiencing symptoms of psychosis^18^. However, growing evidence have suggested the MBIs can be safe and effective with some modification in patients with psychosis^15,19^. One meta-analysis study reported that MBIs were moderately effective in pre-post studies but found to have small-to-moderate effect sizes when studies included a comparison group^20^. They were moderately effective in reducing negative and affective symptoms and in increasing functioning and quality of life. For positive symptoms, results suggested smaller effects.

However, mindfulness requires conscious effort and can be difficult to maintain, particularly for patients with psychosis having cognitive impairment. In recent years there has been an interest in using VR to support mindfulness practice. As VR is a very attention-grabbing technology in terms of providing a set of images and sounds of real-life situations and can be experienced without the constraints of time and space, VR-based mindfulness (VBM) may help patients focus their attention on achieving a state of mindfulness more easily and more conveniently. Moreover, it can be more attractive and compelling to young people with strong sense of adventure and desire for new technology. The VBM has already demonstrated positive outcomes in patients with depression^21^, panic disorder^22^, post-traumatic stress disorder^23^, methamphetamine use disorder^24^, borderline personality disorder^25^, severe burn^26^, and spinal cord injury^27^. In addition, VR-based guided meditation was found to have beneficial effect on heart rate variability^28^. Nevertheless, to the best of our knowledge, no study was undertaken in the field of psychosis using VBM. This stress the need of conducting a pilot study in psychosis in terms of feasibility of the study protocol, acceptability of the VBM, selection of the most appropriate primary outcome measure and sample size calculation for a full-scale trial.

Therapeutic effects of mindfulness intervention (MI) are known to be related to decentering^29^ Decentering is described as the capacity to take a detached view of one’s thoughts and emotions with a present-focused, nonjudgmental stance^30,31^. Based on this, we hypothesized that VBM would help attain decentering stance about themselves or situations in patients with psychosis which in turn may lead to a reduction of psychopathology. The present pilot study sought to investigate safety and effects of VBM in patients with psychosis. In addition, for an exploratory purpose, physiological measures such as skin conductance level (SCL), hear rate (HR) and RR interval, were collected during the VR intervention in some individuals.

## Methods

### Participants

Patients were recruited from outpatient clinics of psychiatry at four hospitals: Jeonbuk National University Hospital, Chonnam National University Hospital, Inje University Haeundae Paik Hospital, and Kyungpook National University Hospital from April 2020 to Feb 2022. Inclusion criteria were as follows: (a) schizophrenia spectrum disorders (schizophrenia, schizoaffective disorder, schizophreniform disorder) or psychotic disorder not otherwise specified (NOS), (b) between 18 and 59 years of age, and (c) stable outpatients with no change of medication during past 2 months. Diagnoses were established using the criteria of the Diagnostic and Statistical Manual of Mental Disorders fourth edition^32^. Two experienced psychiatrists from each institute participated in the diagnostic evaluation and reached a consensus on final diagnosis through discussion. The exclusion criteria were as follows: (a) IQ ≤ 70, (b) acute, unstable, or severe medical/neurological conditions, or (c) pregnant or lactating.

**Fig. 1:**
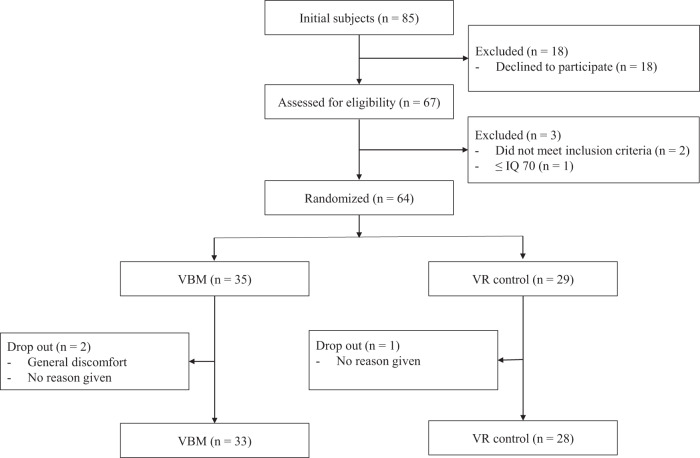
Flow diagram of the study design.

Initially eighty-five individuals were approached but 18 declined to participate. To sixty-seven patients, information about the study objectives and procedure was provided and informed consents were obtained. In the process, three were excluded due to the violation of inclusion/exclusion criteria. The remaining sixty-four patients were randomized to VBM or VR control group within blocks such that similar numbers were assigned to each group. During the trial, three were dropped out (Fig. 1). The authors assert that all procedures contributing to this work comply with the ethical standards of relevant national and institutional Human Experimental Commissions and the 1975 Declaration of Helsinki as amended in 2008. All procedures involving human subjects/patients were approved by Ethics Jeonbuk National University Hospital Committee (approval number CUH 2020-01-042). Trial was registered at the Clinical Research Information Service (KCT0007718).

### Measures

Data on sociodemographic variables (sex, age and education), clinical data and medications were collected. The clinical data included duration of untreated psychosis (DUP) and duration of illness (DI). The total daily dose of antipsychotics at the time of baseline assessment was converted to chlorpromazine (CPZ) dose equivalents based on a defined daily dose^33^. For the evaluation of psychopathology, the Positive and Negative Syndrome Scale^34^ (PANSS), Psychotic Symptom Rating Scale-Delusions & Auditory hallucinations^35^ (PSYRATS-D and PSYRATS-AH) and Columbia-Suicide Severity Rating Scale^36^ (C-SSRS) were performed. The raters were psychiatrists with at least 2-year experience on these measures and blind to the type of intervention randomized to the patients. For self-rating scale, the Experiences Questionnaire (EQ) measuring decentering^37^, Beck Depression Inventory^38^ and Motivation and Pleasure Scale-Self Report^39^ were administered. The MAP-SR is a 15-item scale measuring the motivation and pleasure domain of negative symptoms rated on a 5-point Likert scale. Based on the previous studies demonstrating effects of decentering in depression^40–42^, we considered the EQ as a primary outcome measure. All measures were performed within one-week before and after the intervention. Safety was operationalized as no triggering of significant levels of simulator or VR sickness, and no adverse experiences in the following week. VR sickness refers to symptoms similar to motion sickness (e.g. nausea, dizziness). VR sickness was evaluated using the Simulator Sickness Questionnaire (SSQ)^43^. This was surveyed right after the first and final sessions. Only limited individuals participated in this survey because of its late inclusion in the research protocol and subsequently late IRB approval.

### VR-based mindfulness and VR control

**Fig. 2:**
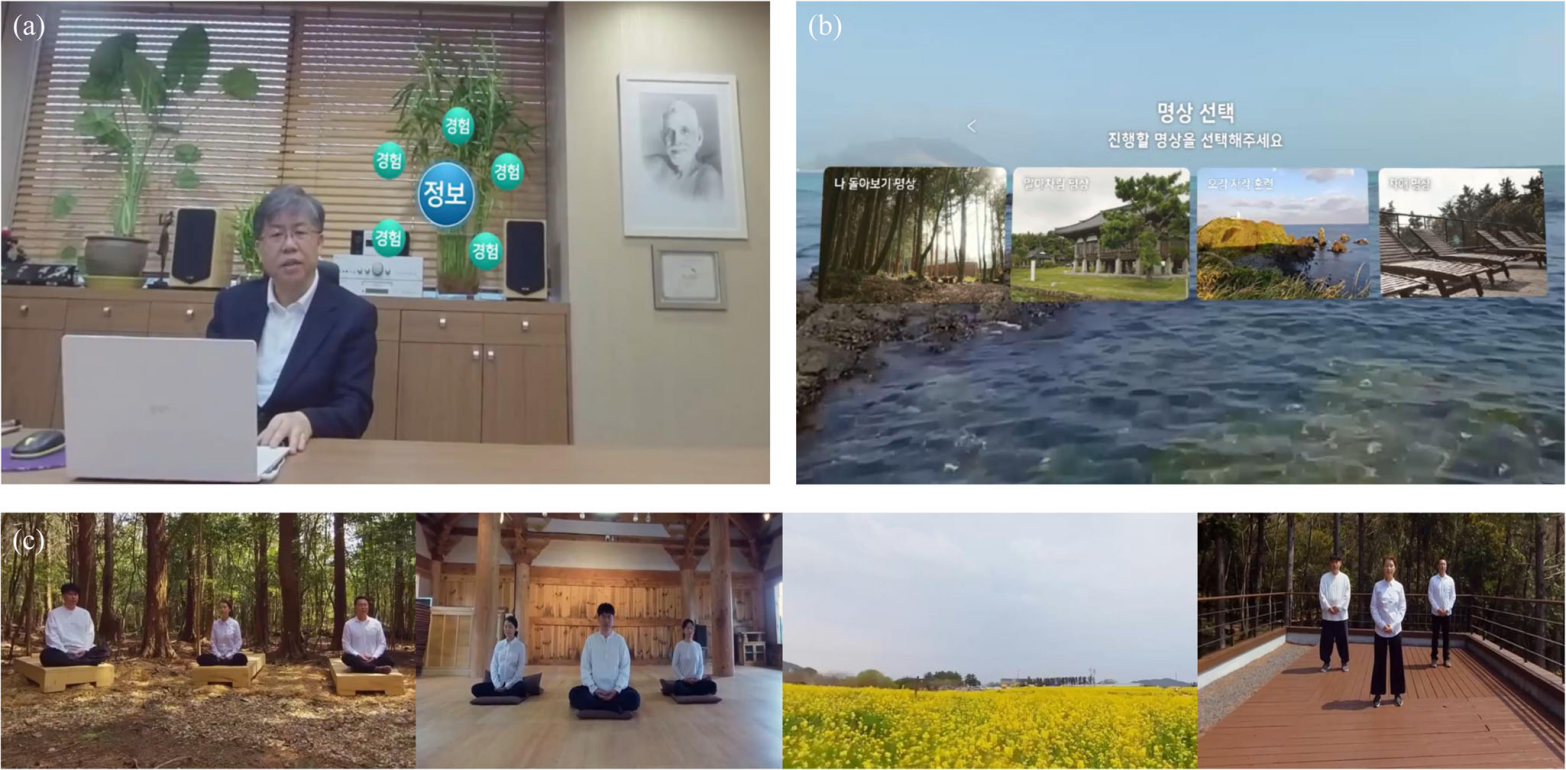
Screenshots of examples of virtual reality based mindfulness interventions. **a** Educational session, **b** a screen displaying options of the meditations and **c** four types of meditations (in order from left to right, looking back at myself meditation, awareness meditation, five-sense awareness meditation and loving-kindness meditation).

VBM was developed using 360 and 3 D camera. It consists of educational (4 min) and therapeutic (5–8 min) videos. The educational video has two parts. The first one is about general introduction of mindfulness and was used in the first four therapeutic sessions. It explains the origin of suffering (attachment and aversion) and emphasizes the importance of strengthening self-monitoring and self-perception to be aware of it. As a first step, focusing on breathing is explained. The second one is about reemphasizing breathing meditation to calm one’s mind, concept of decentering, truth of impermanence and steady practice at home. This video was used in the last four therapeutic sessions. The therapeutic video has four different meditations such as five-sense awareness meditation, awareness meditation, looking back at myself meditation and loving-kindness meditation during which related instructions were given intermittently. The first two types of meditation are to enhance awareness of body sensation and internal thoughts/feelings and the third to improve non-reactive self-related processing^44^. The last one was included based on the recent interest in loving-kindness meditation as an extension of mindfulness constructs^45,46^. Educational material and guiding instructions during therapeutic sessions were developed and recorded by psychiatrists who has full experience and expertise in mindfulness (YH Park and YC Chung). To be tailored to patients with psychosis, formats were designed with short practice time, more guidance and short silence^47^. For shooting meditation videos, experts were recruited and recorded at different places in Jeju island, Korea. For VR control (10 min), 3D videos of nature scenes were selected from Google sites. The nature scenes with calm and relaxing contents were only selected. Screenshots of examples of VR-based MI are shown in Fig. 2. To experience VR, a head-mounted display (Oculus Rift CV1, Reality Labs, Menlo Park, United States) was used. The Head-mounted Display (HMD) has a 90 Hz refresh rate and a 110° field of view with high resolution (1080 × 1200). The VR environments were generated using the Unity engine (Unity Technologies, San Francisco, USA).

A range of physiological stress measures was taken during the VR exposure during the 1st and 8th sessions. Skin conductance level (SCL) was recorded using two gold coated flat and dry surface electrodes (about Ø = 10 mm) that were covered with isotonic electrode gel and placed on the forehead. The sampling rate was 100 Hz. Skin conductance was expressed as mean skin conductance level (μS) between the different data points. Heart rate (HR) was recorded using an earlobe-pulse-plethysmograph (FNIKorea Co., Ltd, Gwacheon, Korea). The sampling rate was 256 Hz. The time intervals between consecutive heart beats, RR intervals, were also measured. For VBM, physiological measures were collected during one educational and two therapeutic video exposures respectively. For VR control, physiologic measures were obtained one time during the whole exposure. Only limited individuals participated in measuring physiological signals because of the same reason with the VR sickness survey.

### Procedures

Participants were randomized to VBM or VR control group via a web-based system (https://www.randomizer.org/). Raters and statistician were blind to group allocation. During the trial, discussion about the cases with names or other potentially identifying information was strictly prohibited. Regardless of the intervention assigned, they received 8 weekly sessions, each lasting 30 min at the digital therapy room of hospital. For every session, they randomly chose two different meditations or nature videos. After application of the equipment for the physiological measurements, participants put on the HMD and experienced the sessions. After the sessions, experiences and any adverse events were discussed. Especially to the participants receiving VBM, core principles of mindfulness and steady practice at home were re-emphasized. During the trial, treatment as usual (supportive psychotherapy, psychoeducation and maintained pharmacotherapy) was provided to all participants.

### Statistical analysis

On the basis of the existing literature on mindfulness or decentering, a small to medium effect size (0.35) was expected^48,49^. A power analysis indicated that 44 subjects per group would give adequate power (0.80) to detect an effect of this size. Group characteristics at baseline between the two groups were compared with a two-sample *t* test or Chi-square test. A 2 × 2 mixed design was employed, comprising two factors with two levels each. The first factor was intervention type (VBM and VR control), and the second factor was time points (pre- and post-intervention). Effects on clinical variables were verified using two-way repeated measure (RM) ANCOVA and paired *t* test for between group and within group comparisons respectively. Because of exploratory nature of the study, multiple comparison was not controlled. For VR sickness, the McNemar test or Chi-square test was performed. One-way RM ANOVA was performed for SCL, HR and RR intervals collected during six time periods (one educational and two meditation videos in the first and 8th sessions) in VBM group. Two-way RM ANOVA was conducted to examine effects of VR interventions on three physiological measures between the VBM and VR control groups. Statistical analyses were performed using R Statistical Software (version 4.3.0, R Foundation for Statistical Computing, Vienna, Austria). All statistical analyses were two-sided, and *p* < 0.05 was considered statistically significant.

## Results

There were no significant differences in demographic and clinical characteristics of the participants between the two groups (Table 1). The two-way RM ANCOVA showed significant group x time interaction only for feelings and motivation about close, caring relationships of the MAP-SR (*p* = 0.027). The post-hoc tests demonstrated a significant difference between the two groups at baseline (*p* = 0.015) but not at the endpoint (*p* = 0.48). For within group comparison, most of subscale scores of the PSYRATS-D were changed significantly in VBM group, and total score and subscale scores of the PANSS changed significantly in both VBM and VR control groups. The BDI score changed only in VR control group (*P* = 0.01) whereas score of the EQ differed only in VBM group (*P* = 0.03) (Table 2).

**Table 1:** Demographic and clinical characteristics of participants.

Characteristics	VBM (*n* = 33)	VR control (*n* = 28)	*P* value
Sex			1
Male	15(45.45%)	13(46.43%)	
Female	18(54.55%)	15(53.57%)	
Age (years)	32.94 ± 11.00	31.32 ± 10.37	0.559
Education (years)	13.73 ± 2.07	13.77 ± 1.76	0.935
DUP (months)	15.47 ± 27.68	15.24 ± 20.81	0.971
DI (months)	114.48 ± 104.59	107.00 ± 107.81	0.785
Diagnosis			0.352
Schizophrenia	28(84.85%)	21(75%)	
Schizophreniform disorder	1(3.03%)	1(3.57%)	
Schizoaffective disorder	1(3.03%)	3(10.71%)	
Psychotic disorder NOS	3(9.09%)	1(3.57%)	
Delusional disorder	0(0%)	2(7.14%)	
Chlorpromazine equivalent dose (mg)	508.75 ± 385.86	399.46 ± 359.35	0.260

*DUP* Duration of untreated psychosis, *DI* duration of illness, *VBM* virtual reality-based mindfulness intervention.

**Table 2:** Comparison of clinical and self-rating scales between the VBM and VR control groups.

Scales	VBM (*n* = 29–33)				VR control (*n* = 25–28)				Main effect				Group × Time interactions	
									Group		Time			
	Before 1st session	After 8th session	Change	P^a^	Before 1st session	After 8th session	Change	P^a^	F	P^b^	F	P^b^	F	P^b^
EQ	29.17 (7.81)	30.86 (7.02)	1.69 (3.63)	0.029	31.60 (5.56)	32.24 (7.77)	0.64 (5.38)	0.557	0.33	0.569	3.88	0.052	0.73	0.394
K-PSYRATS-D														
Total	5.52 (7.21)	4.33 (6.02)	–1.18 (2.46)	0.009	4.61 (5.67)	4.11 (5.43)	–0.50 (1.80)	0.152	1.01	0.317	11.06	0.001	1.69	0.196
Amount of preoccupation	0.91 (1.18)	0.73 (1.01)	–0.18 (0.46)	0.032	0.82 (1.09)	0.71 (1.05)	–0.11 (0.50)	0.264	0.25	0.620	6.32	0.013	0.40	0.527
Duration of preoccupation	1.09 (1.40)	0.79 (1.11)	–0.30 (0.68)	0.016	0.75 (0.89)	0.71 (0.90)	–0.04 (0.43)	0.663	1.65	0.202	6.74	0.011	3.68	0.058
Conviction	1.06 (1.46)	0.94 (1.39)	–0.12 (0.48)	0.160	1.18 (1.44)	1.11 (1.45)	–0.07 (0.38)	0.326	0.27	0.607	3.11	0.080	0.20	0.657
Disruption	0.61 (0.83)	0.45 (0.67)	–0.15 (0.36)	0.023	0.43 (0.50)	0.36 (0.49)	–0.07 (0.38)	0.326	0.33	0.565	10.66	0.001	1.29	0.259
Amount of distress	1.09 (1.53)	0.79 (1.17)	–0.30 (0.68)	0.016	0.82 (1.19)	0.68 (0.98)	–0.14 (0.52)	0.161	0.03	0.858	2.79	0.097	0.18	0.674
Intensity of distress	0.76 (1.03)	0.64 (0.96)	–0.12 (0.48)	0.160	0.61 (0.92)	0.54 (0.84)	–0.07 (0.47)	0.424	0.11	0.738	6.76	0.011	0.82	0.368
K-PSYRATS-AHs														
Total	9.31 (11.19)	8.12 (11.07)	–1.19 (4.60)	0.155	3.08 (6.56)	1.84 (5.06)	–1.24 (4.11)	0.144	0.76	0.386	4.56	0.035	0.00	0.963
Frequency	1.12 (1.39)	0.91 (1.35)	–0.22 (0.71)	0.090	0.36 (0.91)	0.16 (0.62)	–0.20 (0.65)	0.134	0.84	0.360	5.96	0.016	0.01	0.914
Duration	0.75 (1.02)	0.66 (0.94)	–0.09 (0.47)	0.263	0.32 (0.75)	0.16 (0.62)	–0.16 (0.47)	0.103	1.82	0.180	4.31	0.040	0.31	0.580
Location	0.66 (0.94)	0.59 (0.87)	–0.06 (0.80)	0.662	0.24 (0.52)	0.16 (0.62)	–0.08 (0.40)	0.327	0.94	0.336	0.71	0.401	0.01	0.917
Loudness	0.72 (0.99)	0.62 (1.01)	–0.09 (0.47)	0.263	0.32 (0.63)	0.24 (0.66)	–0.08 (0.70)	0.574	0.19	0.664	1.35	0.248	0.01	0.928
Amount of negative content	0.88 (1.29)	0.75 (1.19)	–0.12 (0.55)	0.211	0.40 (0.91)	0.36 (0.91)	–0.04 (0.20)	0.327	0.04	0.833	2.42	0.123	0.56	0.456
Degree of negative content	0.56 (0.76)	0.50 (0.76)	–0.06 (0.44)	0.423	0.16 (0.47)	0.04 (0.20)	–0.12 (0.44)	0.185	1.40	0.239	0.05	0.828	0.47	0.495
Amount of distress	1.31 (1.64)	1.00 (1.48)	–0.31 (0.97)	0.077	0.36 (0.91)	0.20 (0.71)	–0.16 (0.62)	0.212	0.80	0.372	0.44	0.511	0.09	0.762
Intensity of distress	0.83 (1.11)	0.77 (1.10)	–0.05 (0.53)	0.561	0.23 (0.59)	0.13 (0.46)	–0.10 (0.32)	0.134	0.73	0.394	4.90	0.029	0.00	0.964
Belief re-origin of voices	0.84 (1.27)	0.81 (1.28)	–0.03 (0.74)	0.813	0.28 (0.79)	0.20 (0.71)	–0.08 (0.40)	0.327	1.90	0.171	2.90	0.091	0.04	0.834
Disruption	0.84 (1.08)	0.72 (1.05)	–0.12 (0.49)	0.161	0.20 (0.50)	0.08 (0.40)	–0.12 (0.33)	0.083	2.57	0.112	2.56	0.113	0.27	0.604
Controllability	0.66 (0.94)	0.56 (0.88)	–0.09 (0.59)	0.374	0.16 (0.37)	0.04 (0.20)	–0.12 (0.33)	0.083	0.34	0.561	5.56	0.020	0.53	0.469
PANSS														
Total	48.68 (11.44)	43.03 (9.81)	–5.65 (4.96)	<0.001	47.93 (9.55)	43.33 (10.16)	–4.59 (5.27)	<0.001	0.50	0.482	62.58	<0.001	0.65	0.422
Positive symptoms	11.71 (3.61)	10.58 (3.12)	–1.13 (1.71)	<0.001	12.70 (4.48)	11.11 (3.69)	–1.59 (1.87)	<0.001	0.26	0.613	38.55	<0.001	1.14	0.288
Negative symptoms	11.35 (3.92)	11.74 (3.63)	0.39 (3.45)	0.537	10.81 (2.99)	12.56 (4.73)	1.74 (4.05)	0.034	1.45	0.232	4.51	0.036	1.99	0.162
General psychopathology	25.61 (5.93)	22.16 (5.01)	–3.45 (3.24)	<0.001	24.41 (4.70)	22.44 (5.39)	–1.96 (3.76)	0.016	1.78	0.186	38.67	<0.001	2.80	0.097
BDI	11.39 (7.90)	10.84 (8.70)	–0.55 (6.66)	0.553	8.64 (7.12)	6.68 (7.30)	–1.96 (3.40)	0.009	1.70	0.195	2.68	0.105	0.95	0.332
Severity of suicidal ideation^c^	0.68 (1.13)	0.23 (0.61)	–0.45 (1.14)	0.076	0.79 (1.27)	0.47 (0.96)	–0.32 (1.11)	0.230	0.53	0.471	6.88	0.011	0.22	0.643
MAP-SR														
Total	27.16 (14.12)	26.59 (12.40)	–0.56 (9.71)	0.837	33.16 (9.47)	31.32 (12.02)	–1.84 (9.32)	0.333	0.05	0.825	0.85	0.359	0.27	0.604
Social pleasure	5.56 (3.26)	5.41 (3.20)	–0.16 (2.58)	0.734	6.60 (2.35)	6.52 (2.45)	–0.08 (2.60)	0.886	0.54	0.466	0.14	0.708	0.01	0.908
Recreational or work pleasure	5.91 (3.50)	5.19 (3.65)	–0.72 (2.62)	0.131	6.24 (2.44)	6.24 (2.74)	0.00 (2.10)	0.965	1.60	0.209	1.66	0.200	1.30	0.257
Feelings and motivations about close, caring relationships	5.09 (3.14)	5.72 (2.34)	0.62 (2.41)	0.118	7.12 (2.86)	6.16 (3.16)	–0.96 (3.38)	0.169	0.42	0.519	0.04	0.841	5.06	0.027
Motivation and effort to engage in activities	10.78 (5.59)	10.28 (5.56)	–0.50 (4.65)	0.508	13.20 (4.44)	12.40 (5.41)	–0.80 (4.37)	0.299	0.15	0.702	1.17	0.281	0.07	0.799

*EQ* Experience Questionnaire decentering, *K-PSYRATS* Korean Version of the Psychotic Symptom Rating Scales, *PANSS* Positive and Negative Syndrome Scale, *BDI* Beck Depression Inventory,

*MAP-SR* The Motivation and Pleasure Scale-Self Rating items, *VBM* Virtual Reality-based Mindfulness Intervention.

^a^Paired *t* test.

^b^two-way repeated measure ANCOVA.

^c^Mean score of suicidal ideation frequency and duration in the Columbia Suicide Severity Rating Scale. (*n* = 22 (VBM), 19 (VR control)).

For VR sickness, most common symptoms in VBM group were difficulty with visual focus [10/13 (77 %)], blurred vision [9/13 (69%)] and eye fatigue [9/13 (69%)] whereas in the control group, blurred vision [10/15 (67%)], general discomfort [9/15 (60%)] and difficulty with visual focus [8/15 (53%)]. In the VBM group, percentage of fullness of head decreased after 8th session relative to after 1st session (*P* = 0.03). In the VR control group, opposite finding was found (*P* = 0.046). There were no significant differences in the incidence of symptoms of VR sickness between the two groups at both after 1st session and after 8th session (Table 3). The severity of symptoms of VR sickness was usually less than 2 (moderate) in both groups (Table S1).

**Table 3:** Symptoms of VR sickness reported after 1st and after 8th session.

	VBM (*n* = 13)			VR control (*n* = 15)			*P* value^b^	*P* value^c^
	After 1st session	After 8th session	*P*^a^	After 1st session	After 8th session	*P*^a^		
Blurred vision								
Yes	9(69.23%)	8(61.54%)	0.564	10(66.67%)	8(53.33%)	0.414	1	0.956
No	4(30.77%)	5(38.46%)		5(33.33%)	7(46.67%)			
Burping								
Yes	0(0%)	1(7.69%)	NA	1(6.67%)	1(6.67%)	NA	1	1
No	13(100%)	12(92.31%)		14(93.33%)	14(93.33%)			
Difficulty concentrating								
Yes	6(46.15%)	4(30.77%)	0.157	5(33.33%)	7(46.67%)	0.317	0.761	0.638
No	7(53.85%)	9(69.23%)		10(66.67%)	8(53.33%)			
Difficulty with visual focus								
Yes	10(76.92%)	9(69.23%)	0.317	8(53.33%)	8(53.33%)	1	0.254	0.638
No	3(23.08%)	4(30.77%)		7(46.67%)	7(46.67%)			
Dizziness when eyes closed								
Yes	3(23.08%)	1(7.69%)	0.157	1(6.67%)	0(0%)	NA	0.311	0.464
No	10(76.92%)	12(92.31%)		14(93.33%)	15(100%)			
Dizziness when eyes opened								
Yes	3(23.08%)	1(7.69%)	0.157	1(6.67%)	1(6.67%)	1	0.311	1
No	10(76.92%)	12(92.31%)		14(93.33%)	14(93.33%)			
Eye fatigue								
Yes	9(69.23%)	7(53.85%)	0.157	6(40%)	3(20%)	0.083	0.243	0.114
No	4(30.77%)	6(46.15%)		9(60%)	12(80%)			
Fatigue								
Yes	7(53.85%)	4(30.77%)	0.083	3(20%)	6(40%)	0.180	0.114	0.705
No	6(46.15%)	9(69.23%)		12(80%)	9(60%)			
Fullness of head								
Yes	8(61.54%)	3(23.08%)	0.025	3(20%)	7(46.67%)	0.046	0.063	0.254
No	5(38.46%)	10(76.92%)		12(80%)	8(53.33%)			
General discomfort								
Yes	8(61.54%)	5(38.46%)	0.083	9(60%)	10(66.67%)	0.655	1	0.266
No	5(38.46%)	8(61.54%)		6(40%)	5(33.33%)			
Headache								
Yes	3(23.08%)	2(15.38%)	0.564	2(13.33%)	2(13.33%)	1	0.639	1
No	10(76.92%)	11(84.62%)		13(86.67%)	13(86.67%)			
Increased salivation								
Yes	3(23.08%)	3(23.08%)	1	2(13.33%)	1(6.67%)	0.317	0.639	0.311
No	10(76.92%)	10(76.92%)		13(86.67%)	14(93.33%)			
Nausea								
Yes	2(15.38%)	0(0%)	NA	1(6.67%)	0(0%)	NA	0.583	NA
No	11(84.62%)	13(100%)		14(93.33%)	15(100%)			
Stomach discomfort								
Yes	0(0%)	2(15.38%)	NA	1(6.67%)	1(6.67%)	NA	1	0.583
No	13(100%)	11(84.62%)		14(93.33%)	14(93.33%)			
Sweating								
Yes	2(15.38%)	1(7.69%)	0.317	1(6.67%)	0(0%)	NA	0.583	0.464
No	11(84.62%)	12(92.31%)		14(93.33%)	15(100%)			
Vertigo								
Yes	3(23.08%)	2(15.38%)	0.317	1(6.67%)	1(6.67%)	NA	0.311	0.583
No	10(76.92%)	11(84.62%)		14(93.33%)	14(93.33%)			

*VBM* Virtual Reality-based Mindfulness Intervention.

^a^McNemar test.

^b^Chi-square test for comparison of adverse events after 1st session between two groups.

^c^Chi-square test comparison of adverse events after 8th session between two groups. Data given as *N* (percent%).

We were able to collect physiological measures from 8 and 9 patients allocated to VBM and VR control groups. One-way RM ANOVA showed a significant main effect in time only for HR (F (5,35) = 3.10, *P* = 0.02) (Table S2). In the post-hoc test, significant differences were shown between T1 vs. T3 and T5 (Fig. S1). Two-way RM ANOVA showed a significant main effect in time only for HR (F (1,16) = 8.69, *P* = 0.01) (Table S3). Post-hoc test showed a significant difference of HR in the first session vs. HR in the 8th session only in VBM group (Fig. S2).

## Discussion

VR technology can facilitate mindfulness learning by reducing the interference of distractors from the natural environment and providing a sense of presence in immersive environments. These qualities have great potential to benefit research in that extraneous variables that would otherwise be uncontrollable can be limited. There has been widespread use of VR-based MI in diverse clinical areas but surprisingly no study in psychosis. The present study is the first randomized controlled study exploring safety and effects of VBM in patients with psychosis. The VBM was tolerable but the frequency of VR sickness was high. We could not observe a significant effect of the VBM on the decentering, a primary outcome, between the two groups but on the change of feelings and motivations about close caring relationships in the MAP-SR.

We observed a significant reduction of decentering in the treatment group but not in the control group. Studies have shown that decentering reduced levels of depressive rumination by teaching patients more adaptive ways of relating to their thinking^40^ and was associated with less depressive symptoms^41^ or the lowest rates of relapse of depression in the 18-month follow-up period^42^. However, using keywords such as decentering, psychosis and/or schizophrenia, no studies were found. Instead, several studies investigated “decentration” in patients with schizophrenia^50–52^ along with self-reflectivity, understanding of others’ minds and mastery. It is defined as the ability to see others as having independent motives like theory of mind and could be considered as one component of social cognition whereas decentering is focused on self-awareness. However, we did not observe significant results in group x time interaction and main effects of group and time. In other words, these findings suggest that increased decentering in the treatment group was not enough to produce a significant difference compared to the control group. As the degree of presence could be influential in mindfulness research incorporating VR, causes for this negative finding may be interpreted that the setting or contents of our VBM were not good enough to produce a fully immersive feeling of presence. Or it may be related to a relatively small sample size of the participants.

Interestingly, we found significant reductions of the amount and duration of preoccupation in the PSYRATS-D in the treatment group but not in the control group. Also, between group difference for the duration of preoccupation was a trend toward significance. This may be related to increased decentering in the treatment group. This speculation may be supported by the results of additional correlation analysis that the score of the EQ was negatively associated with the duration of preoccupation ($r$ = –0.43, *P* = 0.01) and amount of preoccupation ($r$ = –0.34, *P* = 0.54) at the post-assessment. Given that effect of MI on positive symptoms is generally small^20^ and scores of the PSYRATS-D in the present study were very low, our finding is encouraging. During the trial, we felt that in order to enhance decentering stance more effectively in psychosis, guiding comments should be modified from typical approach, being aware of one’s own drive or attachment and acceptance of one’s own bad words and behaviors, to a new transformative approach, being aware of one’s own trauma/hurts coming from others drive or attachment and focusing more on self-compassion and self-kindness. This new approach may be more fit with patient mind status fully filled with fear/threatened feelings, anger, shame/guilt or mistrust. Additionally, it is of note that both education before the session and instructions given during the session were general explanations about stress and mindfulness, not mentioning specific symptoms. If we further incorporate specific mindfulness-based education or instruction on how to cope with positive symptoms in the program, this would yield more encouraging results on positive symptoms which need to be tested in future studies. Collectively, our results suggest that VBM is beneficial in enhancing decentering and decreasing preoccupation and distress related to delusion in patients with psychosis. As for the results on the PSYRATS-AH, there were no significant changes in any domains of the PSYRATS-AH after VR-based MI in both treatment and control groups. This indicates that VBM is not effective in reducing AHs even though baseline scores of the PSYRATS-AH in the treatment group were significantly higher compared to the control group. Psychological therapies for voices emphasize a decentered relationship with voices such as an awareness of experiences, maintenance of distance and disidentification from them^53^. It should be noted that in one randomized controlled trial with large sample size, AVATAR therapy for AH involves specifically how to cope with voices such as assertive responding and dialog with therapist^54^. In order to see positive results on AHs, it seems necessary to modify contents of VBM.

For the PANSS, there were no significant results in group x time interaction and main effect of group However, within group comparisons showed significant improvements in total, positive symptom and negative symptom scores of the PANSS in both groups. These findings suggest that both interventions are effective in reducing psychopathologies but effect of VBM is not superior to that of the VR control. One important controversial issue is what is the best and optimal control condition for VBM. Other studies adopted a wait-list approach, VR mental relaxation, or supportive counseling. We believe that our VR control condition is optimal in that every setting is the same except the contents of the VR. This may have contributed to the negative findings for between-group differences. Importantly, we observed a significant group x time interaction in the feelings and motivations about close caring relationships between the two groups. Considering that there were no within-group differences in both groups, this finding may be driven by the sum of little increase in the treatment group and little decrease in the control group. The decrease of the feelings and motivations about close caring relationships in the control group may be related to the increase in the negative symptoms in the control group. In other words, these findings collectively suggest that VBM might be beneficial in preventing aggravation of feelings and motivations about close caring relationships or negative symptoms compared to VR control. This possibility should be explored in future studies with a larger sample.

Regarding safety, most of the participants reported various symptoms of VR sickness: the most common symptoms in the treatment group were difficulty with visual focus, blurred vision and eye fatigue and in the control group, blurred vision, general discomfort and difficulty with visual focus. However, severity of symptoms was mostly mild level. In addition, there were no significant differences in the frequency of symptoms between the two groups. These findings indicate that VBM and VR control can be carried out safely in patients with psychosis which is in line with other studies in psychosis^9,54^. The safe profile of VR-based programs may be related to very low attrition rates (3.4 or 5.7%) in the two groups. This could be considered as a strength of using VR-based programs, especially in young individuals with psychosis. Analysis of physiological measures revealed that there were significant differences only in HR over time in the VBM group. Although the effects were not marked enough to produce a significant group difference, these findings suggest that VBM could decrease autonomic activity over time in patients with psychosis.

There are limitations to the study. First, as sample size was relatively small, type II error may exist. In addition, DI and education of the participants were relatively longer and higher. These factors limit the generalizability of our findings. Second, the duration of each session was about 30 min which is a little shorter^55,56^ or similar^57^ when compared to other studies. Third, as a short dialog with therapists was carried out after each session in the treatment group to enhance understanding of MI, the quality of therapists may have affected the results. Even though we had introductory online meetings about therapist roles, its control remains crucial in the multi-sites study. Fourth, we did not collect data on prior experiences of VR which may affected results differently. Fifth, the long-term effects of VBM should be explored further. Albeit these caveats, strengths of our study are that this is the first RCT using VBM in patients with psychosis and VR control was optimally designed.

In summary, we did not observe significant differences in decentering between the two groups but a significant difference in group x time interaction for feelings and motivations about close caring relationships of the MAP-SR. In addition, patients randomized to VBM showed increased decentering and decreased amount and duration of preoccupation in the PSYRATS-D although their changes were not enough to produce between-group difference. The frequency of VR sickness was high but its severity was mild and acceptable. These results suggest that VBM was not more effective in reducing decentering and psychiatric symptoms than VR control but its adversity was modest. Given the feasibility of the research design and procedures, acceptability and tolerability of the VBM, and possible type II error, it warrants a full-scale trial with a larger sample size and more rigorous methodology.

### Supplementary information

2022-Effects of VTm in psychosis-supplementary material
