# Supplementary material for: Effects and safety of virtual reality-based mindfulness in patients with psychosis: a randomized controlled pilot study
Source: Schizophrenia (Heidelb). 2023 Sep 13;9(1):57. doi: 10.1038/s41537-023-00391-8 (PMC10499950; doi:10.1038/s41537-023-00391-8)
Supplement: Supplementary file 1 — 2022-Effects of VTm in psychosis-supplementary material [file 41537_2023_391_MOESM1_ESM.docx]

Table S1. Comparison of scores for symptoms of VR sickness

|  |  | VBM | | | | |  | VR control | | | | |  | p-value^b^ | p-value^c^ |
| --- | --- | --- | --- | --- | --- | --- | --- | --- | --- | --- | --- | --- | --- | --- | --- |
|  |  | N | Before 1^st^ session | After 8^th^ session | Change | p^a^ |  | N | Before 1^st^ session | After 8^th^ session | Change | p^a^ |  |  |  |
| Blurred vision |  | 7 | 1.71 ± 0.76 | 1.57 ± 0.53 | -0.14 ± 0.69 | .773 |  | 6 | 1.50 ± 0.55 | 1.33 ± 0.52 | -0.17 ± 0.41 | .363 |  | .577 | .942 |
| Burping |  | 0 | NA | NA | NA | NA |  | 1 | 1.00 ± 0.00 | 1.00 ± 0.00 | 0.00 ± 0.00 | NA |  | NA | NA |
| Difficulty concentrating |  | 4 | 1.25 ± 0.50 | 1.50 ± 1.00 | 0.25 ± 0.50 | .391 |  | 4 | 1.25 ± 0.5 | 1.75 ± 0.96 | 0.50 ± 0.58 | .182 |  | 1 | .537 |
| Difficulty with visual focus |  | 9 | 1.67 ± 0.71 | 1.44 ± 0.73 | -0.22 ± 1.09 | .710 |  | 4 | 1.50 ± 0.58 | 1.75 ± 0.50 | 0.25 ± 0.50 | .391 |  | .689 | .434 |
| Dizziness when eyes closed |  | 1 | 1.00 ± 0.00 | 2.00 ± 0.00 | 1.00 ± 0.00 | NA |  | 0 | NA | NA | NA | NA |  | NA | NA |
| Dizziness when eyes opened |  | 1 | 1.00 ± 0.00 | 1.00 ± 0.00 | 0.00 ± 0.00 | NA |  | 0 | NA | NA | NA | NA |  | NA | NA |
| Eye fatigue |  | 7 | 1.57 ± 0.79 | 1.14 ± 0.38 | -0.43 ± 0.98 | .345 |  | 3 | 1.33 ± 0.58 | 1.67 ± 0.58 | 0.33 ± 0.58 | .423 |  | .654 | .251 |
| Fatigue |  | 4 | 1.00 ± 0.00 | 1.25 ± 0.50 | 0.25 ± 0.50 | .391 |  | 2 | 1.50 ± 0.71 | 1.50 ± 0.71 | 0.00 ± 1.41 | 1 |  | .500 | .745 |
| Fullness of head |  | 3 | 1.00 ± 0.00 | 1.67 ± 0.58 | 0.67 ± 0.58 | .184 |  | 3 | 1.67 ± 1.15 | 1.67 ± 0.58 | 0.00 ± 1.00 | 1 |  | .423 | .374 |
| General discomfort |  | 5 | 1.00 ± 0.00 | 1.00 ± 0.00 | 0.00 ± 0.00 | NA |  | 7 | 1.43 ± 0.53 | 1.29 ± 0.49 | -0.14 ± 0.69 | .773 |  | .108 | .604 |
| Headache |  | 1 | 1.00 ± 0.00 | 2.00 ± 0.00 | 1.00 ± 0.00 | NA |  | 1 | 1.00 ± 0.00 | 1.00 ± 0.00 | 0.00 ± 0.00 | NA |  | NA | NA |
| Increased salivation |  | 2 | 1.50 ± 0.71 | 2.00 ± 1.41 | 0.50 ± 2.12 | .795 |  | 1 | 1.00 ± 0.00 | 2.00 ± 0.00 | 1.00 ± 0.00 | NA |  | .667 | NA |
| Nausea |  | 0 | NA | NA | NA | NA |  | 0 | NA | NA | NA | NA |  | NA | NA |
| Stomach discomfort |  | 0 | NA | NA | NA | NA |  | 1 | 1.00 ± 0.00 | 1.00 ± 0.00 | 0.00 ± 0.00 | NA |  | NA | NA |
| Sweating |  | 1 | 1.00 ± 0.00 | 2.00 ± 0.00 | 1.00 ± 0.00 | NA |  | 0 | NA | NA | NA | NA |  | NA | NA |
| Vertigo |  | 0 | 1.50 ± 0.71 | 1.50 ± 0.71 | 0.00 ± 0.00 | NA |  | 1 | 1.00 ± 0.00 | 1.00 ± 0.00 | 0.00 ± 0.00 | NA |  | .667 | NA |

^a^Paired t-test, ^b^t-test for comparison of baseline data between two groups, ^c^t-test for comparison of change values between two groups; Data given as Mean$\pm$SD.

*VBM* Virtual Reality-based Mindfulness Intervention.

**Table S2.** One-way repeated measures ANOVA for heart rate over time in patients allocated to VBM (n=8).

|  | Sum Sq | df | Mean Sq | F | p |
| --- | --- | --- | --- | --- | --- |
| Time | 500.2 | 5 | 100.0 | 3.095 | 0.0204 |


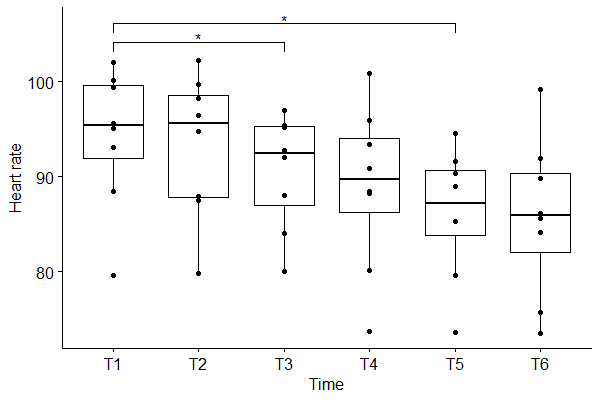


**Figure S1.** Heart rate during the first session (T1 [first educational video], T2, and T3 [two meditation videos]) and 8th session (T4 [second educational video], T5 and T6 [two meditation videos]) in patients receiving VBM.

*p-value=0.048 (T1 and T3), 0.044 (T1 and T5).

**Table S3.** Two-way repeated measures ANOVA for heart rate over time between the two groups (n=8-9).

|  | F | p |
| --- | --- | --- |
| Group | $F_{1,16}$= 2.232 | 0.1560 |
| Time | $F_{1,16}$= 8.690 | 0.0100 |
| Group*Time | $F_{1,16}$= 0.305 | 0.5888 |


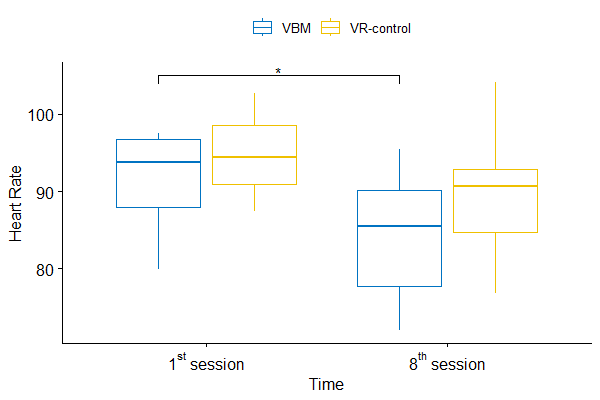


**Figure S2.** Comparison of heart rate in the first and 8th session between VBM and VR control groups

Note: Heart rates in the first and 8th session in the VBM group were obtained by averaging heart rates in one educational and two meditation videos in each session.

*p-value=0.051
